# Determinants of Influenza Vaccination Uptake Among Elderly Residents in Nursing Homes: A Cross-Sectional Analysis of Barriers and Strategic Implications

**DOI:** 10.3390/vaccines14040302

**Published:** 2026-03-27

**Authors:** Ye Qiu, Hui Qiao, Yanting Yang, Tingting Jiang, Jin Zhang, Yuanping Wang

**Affiliations:** Shanghai Pudong New Area Center for Disease Control and Prevention (Shanghai Pudong New Area Health Supervision Institute), Shanghai 200136, China; pdcdcxd_qy@163.com (Y.Q.);

**Keywords:** influenza, vaccination, coverage, willingness, older people, nursing homes

## Abstract

Background: Nursing homes are congregate settings for elderly individuals where infectious diseases can easily spread. The elderly are at high risk of contracting and dying from influenza, and the most effective way to prevent this is to receive the influenza vaccine. Methods: This study conducted a cross-sectional survey of elderly people in nursing homes to investigate the occurrence of influenza symptoms during the 2024–2025 flu season, as well as vaccination status and reasons for receiving or not receiving the vaccine. Bivariate logistic regression was used to determine the factors influencing the vaccination rate. Results: Of the 1024 elderly people who participated in the survey, 25.39% reported experiencing flu-related symptoms in the previous flu season. While 16.21% of the elderly expressed willingness to receive vaccination, only 5.57% actually received it. Influenza vaccination was positively correlated with educational attainment (aOR 3.800, 95% CI 1.480–9.758 for middle school; aOR 5.138, 95% CI 1.738–15.191 for high school), monthly household income (aOR 0.216, 95% CI 0.072–0.644 for >8000), ability for self-care (aOR 0.269, 95% CI 0.123–0.591), and the scale of the nursing home (aOR 9.033, 95% CI 1.531–53.305 for 151–299; aOR 2.629, 95% CI 1.359–5.084 for ≥300). Willingness to receive the influenza vaccination was positively correlated with an unhealthy health status (aOR 0.398, 95% CI 0.204–0.779), symptoms of influenza (aOR 2.730, 95% CI 1.861–4.007), nursing home location (aOR 1.537, 95% CI 1.099–2.941 for outer suburbs), and the scale of the nursing home (aOR 1.991, 95% CI 1.154–3.435 for 151–299; aOR 2.158, 95% CI 1.374–3.390 for ≥300). Most elderly people who received the vaccine believed that vaccination could effectively prevent flu and that it could reduce the risk of complications, the rest were not vaccinated due to concerns about adverse reactions, mobility issues, or the distance to vaccination sites. Conclusions: Low awareness of flu vaccines and physical inability to travel to vaccination sites may be potential barriers to receiving the flu vaccine. It is worrying that the influenza vaccination rate is low among the elderly in nursing homes in Shanghai. As a result, it is crucial to prioritize targeted monitoring and intervention strategies for vulnerable populations living in collective institutions.

## 1. Introduction

Influenza is an acute respiratory infection caused by influenza viruses that are highly contagious and experience seasonal outbreaks. Its high morbidity and mortality rates pose a major threat to global public health [1,2]. In China, elderly people die from excess mortality caused by influenza-related respiratory diseases, which account for 80% of all respiratory disease deaths [3,4,5]. The elderly are more likely than other age groups to develop severe complications from influenza, putting them at higher risk of serious illness and death [6].

Getting the influenza vaccine can effectively reduce the incidence of influenza among the elderly, as well as related complications, hospitalizations, and deaths. It is the most economical and effective intervention for preventing influenza. The “Healthy China Action (2019–2030)” [7] and the “14th Five-Year Plan” [8] emphasize the importance of vaccines in preventing disease and encourage high-risk groups to get the flu vaccine before the flu season. The Chinese Center for Disease Control and Prevention (China CDC) recommends prioritizing the vaccination of people aged 60 and over [2]. However, influenza vaccines are not included in China’s National Immunization Program (NIP). Most regions in China still rely on self-funding for influenza vaccinations. Only a few cities, such as Beijing [9,10] and Taizhou City in Zhejiang Province [11], provide free or subsidized vaccines to the elderly and young children. Only 2.27% to 17.68% [12,13,14] of the general population has been vaccinated, which is lower than that in the United States (37.44%), Canada (36.91%), and European countries [15].

Italy, Austria, New Zealand, Switzerland, and the Netherlands all have policies that make flu vaccines free or reimbursable for the elderly, which greatly increases the number of elderly people who get vaccinated. In Italy [16], 62.6% of the elderly are vaccinated, including 50.8% of the homebound elderly and 74.0% of the elderly in nursing homes. In Austria [17], New Zealand, Switzerland, and the Netherlands [18], the vaccination rates for the elderly in nursing homes are 19.1%, 78.5%, 65.4%, and 75.2%, respectively.

Shanghai is a major city in eastern China and one of the most developed cities in the world. According to the results of the Seventh National Population Census, the population of Shanghai was 24.87 million in 2020, of which 6.68 million (26.86%) were aged 60 or over, which is higher than the national average of 18.7% [19]. Currently, home-based care cannot meet the needs of an aging society, and more elderly people are choosing to live in nursing homes. We previously conducted a study on influenza vaccination coverage, willingness, and influencing factors in Shanghai after the COVID-19 pandemic [20]. We have not found any analysis of the influenza vaccination situation of elderly people in nursing homes in China.

This study focuses on the elderly in nursing homes in Shanghai’s Pudong New Area, analyzed influenza vaccination coverage and willingness, and identified determinants of vaccine uptake. The findings can provide a scientific basis for the formulation of vaccination strategies and programs for the elderly in nursing homes.

## 2. Materials and Methods

### 2.1. Study Design and Sampling Procedure

We conducted a cross-sectional study on influenza vaccination status among the elderly in nursing homes in Pudong New Area from March to April 2025. We selected 20 nursing homes for investigation using the means of surface with non-homogeneity (MSN), and considered multiple characteristics, including:

Geographical location (urban areas, urban-rural fringe zones, outer suburbs);

Institutional grade (from lowest to highest, the levels are: unrated, level 1, level 2, and level 3, based on a comprehensive assessment of management, funding, etc.);

Nature (public, private);

Scale (large: ≥300 beds; medium: 151–299 beds; small: <150 beds).

The required sample size was estimated: N = [π(1 − π) × Zɑ^2^]/d^2^. According to the influenza vaccination coverage rate (VCR) for the elderly in Shanghai (π = 9.50% [20,21], d = 0.15π, α = 0.05), a sample size of 830 elderly people in nursing homes is required. Considering 10% of questionnaires being invalid, at least 913 people should be surveyed. Each nursing home should randomly select 46 people to participate in the study. (Note: The π value was calculated based on the averages of local influenza VCR during the 2022–2023 [20] and 2018–2019 [21] influenza season.)

The inclusion criteria were: (1) Aged 60 years or older; and (2) residing in the selected nursing homes for at least 6 months. The exclusion criterion was elderly individuals with severe mental illness who could not fully participate in the study.

### 2.2. Data Collection

Face-to-face interviews were conducted at each selected nursing institution by staff trained by professional investigators at the Shanghai Pudong New Area Center for Disease Control and Prevention (PDCDC). The interviews were conducted with the elderly themselves or their children, and data were collected using Questionnaire Star. The vaccination information collected was verified using the Shanghai Immunization Planning Information System (SIPIS).

The questionnaire consisted of three sections: (1) Demographic information, such as gender, age, marital status, educational attainment, number of children, monthly family income, health status (including self-care ability, etc.), and chronic diseases; (2) influenza infection status within the past six months, including fever (temperature ≥ 38 °C) or muscle and joint pain, sore throat, cough, nasal congestion, and other symptoms (whether to report to the infirmary or staff in the nursing home, or whether to go to the hospital for treatment); and (3) influenza vaccination status, willingness to vaccinate, related reasons and influencing factors.

The validity of responses is determined by the completeness of the survey, the logic of the responses, and the time spent reading and answering the survey questions. Questionnaires missing more than 20% of core items (including basic information such as gender and age, whether to vaccinate, etc.) were defined as invalid and excluded from the final analysis. For individual missing items of non-core variables, multiple imputation by chained equations (MICE) was performed to maintain statistical power. The minimum time required to read all questions and provide answers was calculated as 60 s; responses submitted in less than 60 s were considered invalid, as they suggested that respondents had not fully read the questions before answering.

### 2.3. Statistical Analysis

Data collection and analysis were performed using Excel 2016 software and SPSS version 26.0 software (International business machines, Armonk, NY, USA). All variables were categorical and presented as frequencies and percentages.

The chi-square test was used for single-factor analysis of all variables; Fisher’s exact test was applied when the expected frequency in any cell was less than five. *p*-values < 0.05 in the analysis were included as variables for multivariate analysis. Before constructing the regression model, multicollinearity among independent variables was assessed using the variance inflation factor (VIF) and tolerance index. A VIF value < 5 and tolerance > 0.2 were defined as the threshold for no significant multicollinearity, and all variables met this criterion (VIF range: 1.012–1.809).

A binary logistic regression model was constructed to identify factors associated with influenza vaccination uptake. The forward conditional method was used to model, and odds ratios (aORs) with 95% confidence intervals (CIs) were calculated. A *p*-value < 0.05 was considered statistically significant for all analyses.

## 3. Results

### 3.1. Characteristics of Study Population

**Figure 1:**
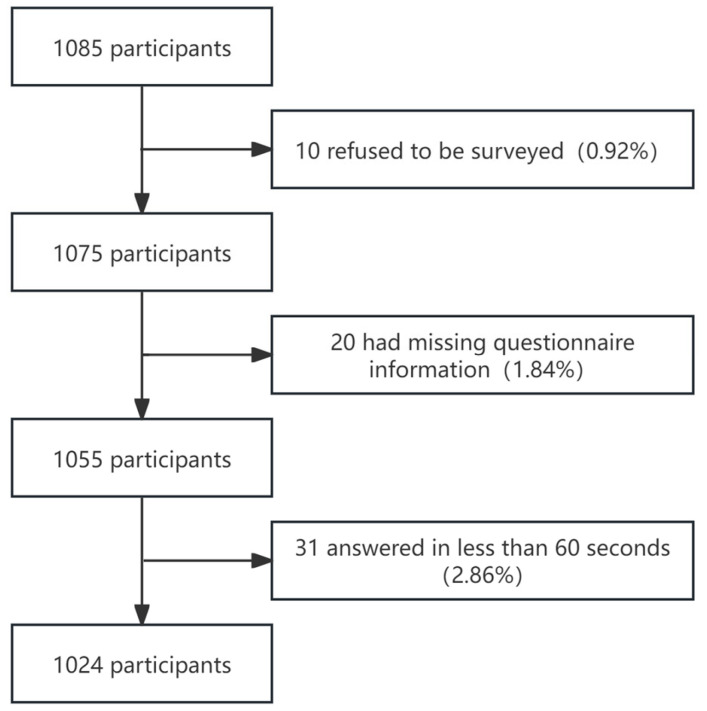
Completion of enrollment survey questions among 1024 elderly nursing home residents recruited in Shanghai, China, 2025.

A total of 1085 elderly individuals participated in the survey—all of the survey responses were completed, and 1024 valid responses were obtained (Figure 1).

The age range of the survey respondents was from 60 to 104 years old, with a median age of 87 years old [(IQR): 82–90]. Females accounted for 70.02% of the respondents. The majority of respondents (85.35%) were local residents. Nearly three-quarters (71.58%) of respondents were divorced or widowed. Nearly two-thirds (64.46%) of the elderly had received primary school education or below. More than half (53.12%) of the elderly were unable to care for themselves, and 51.27% of the elderly had two or more chronic diseases. Furthermore, most of the elderly lived in private (68.26%), suburban (58.30%), and medium-sized (43.46%) nursing homes.

Among the 1024 elderly individuals who participated in the survey, 25.39% reported experiencing flu-related symptoms in the previous flu season. A total of 43.07% of the elderly went to medical institutions, and the pathogen test was positive for influenza. The rest of the elderly in the nursing home were tested with rapid detection kits in the infirmary or were administered drugs after the doctor’s diagnosis.

### 3.2. Influenza Vaccination Coverage Rate and Their Influencing Factors

A total of 16.21% of elderly residents in nursing homes expressed willingness to receive the influenza vaccination, but only 5.57% of them received it.

Analysis of the factors influencing the vaccination of elderly people in nursing homes revealed that vaccination is related to educational attainment, monthly household income, self-care ability, and the location and scale of the nursing home (*p* < 0.05) (Table 1).

Analysis of the factors influencing the willingness of elderly people in nursing homes to be vaccinated revealed that the willingness to be vaccinated is related to the number of children, health status, the presence of flu-like symptoms, and the location and scale of the nursing home (*p* < 0.05) (Table 1).

Educational attainment, monthly household income, self-care ability, and the location and scale of the nursing home were included in the multivariate logistic regression model analysis. Influenza vaccination was positively correlated with educational attainment (aOR 3.800, 95% CI 1.480–9.758 for middle school; aOR 5.138, 95% CI 1.738–15.191 for high school), monthly household income (aOR 0.216, 95% CI 0.072–0.644 for >8000), ability for self-care (aOR 0.269, 95% CI 0.123–0.591), and the scale of the nursing home (aOR 9.033, 95% CI 1.531–53.305 for 151–299; aOR 2.629, 95% CI 1.359–5.084 for ≥300) (Table 2).

Number of children, health status, the presence of flu-like symptoms, and the location and scale of the nursing home were included in the multivariate logistic regression model analysis. Willingness to receive the influenza vaccination was positively correlated with an unhealthy health status (aOR 0.398, 95% CI 0.204–0.779), symptoms of influenza (aOR 2.730, 95% CI 1.861–4.007), nursing home location (aOR 1.537, 95% CI 1.099–2.941 for outer suburbs), and the scale of the nursing home (aOR 1.991, 95% CI 1.154–3.435 for 151–299; aOR 2.158, 95% CI 1.374–3.390 for ≥300) (Table 3).

### 3.3. Reasons for Receiving or Not Receiving the Influenza Vaccine

**Figure 2:**
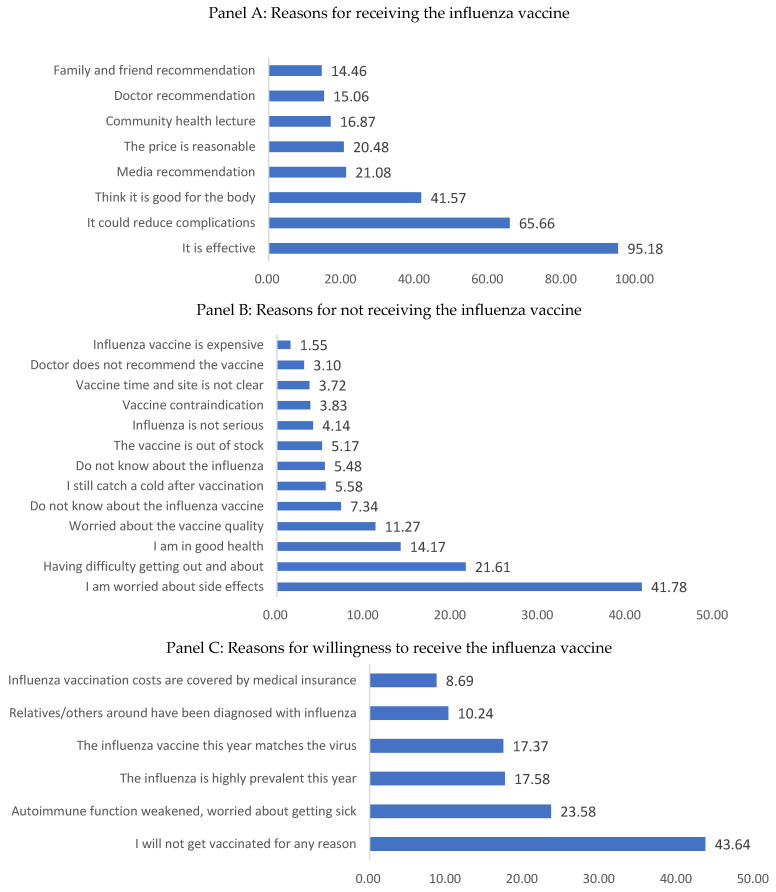
Reasons for elderly individuals in nursing homes receiving/not receiving/willingness to receive the influenza vaccine.

The majority of elderly people who received the vaccine (95.18%) believed that vaccination could effectively prevent influenza. Two-thirds (65.66%) believed that it reduced the risk of complications, and 41.57% considered it beneficial to their health (Figure 2A).

The main reasons for not receiving the influenza vaccine were concerns about adverse reactions (41.78%), difficulty accessing the vaccine due to mobility issues or distance (21.61%), and the belief that they would not contract influenza (14.71%) (Figure 2B).

We also collected reasons for encouraging vaccination, using prompts such as “What would make you want to get the flu vaccine?”. A total of 43.64% of elderly people said that they would never get the flu vaccine for any reason, while 23.58% would get the flu vaccine if their immune system was weakened and they were concerned about falling ill. Furthermore, 7.58% of the elderly said they would get the flu vaccine if they knew that flu was highly prevalent and the risk of infection was high (Figure 2C).

## 4. Discussion

During the flu season, 25.39% of elderly residents in nursing homes develop flu symptoms, which is much higher than the national rate for older individuals [22]. Because of decreased immune function and a greater chronic disease, elderly people are more likely to develop complications, severe sickness, and die unnecessarily after contracting influenza [23,24]. Although 16.21% of elderly people living in nursing homes are willing to receive the flu vaccine, the actual vaccination rate is only 5.57%, which is a significant decrease from the flu vaccination rate among the elderly in 2022 [20]. This decline may be associated with a reduced awareness of vaccination following the COVID-19 pandemic.

Although the VCR is higher than the national average [25], it is still lower than that in some cities, such as Weifang City (54.89%) [26], Guangzhou City (19.59%) [27], and Jiaozuo City (10.25%) [28]. It is also lower than the rates in other countries, including Spain (57.0–58.5%) [29], South Africa (32.3%) [30], Korea (42.4%) [31], and the Czech Republic (62.2%) [32], falling well below the target recommended by the World Health Organization (WHO). The WHO has explicitly identified older people as a priority group for influenza vaccination and recommends that all individuals aged 65 and above receive the flu vaccine annually [33,34,35]. Currently, the VCR is too low to establish an effective immune barrier for elderly people living in nursing homes. Studies have shown that providing free influenza vaccinations to older people is highly cost-effective [36]. For instance, after Beijing began offering free flu vaccines to elderly people in 2007, the vaccination coverage rate among the older population increased to 50% by 2015 [37].

This study found that higher education and income were protective factors for receiving the flu vaccine among nursing home residents, similar to other studies [38,39,40,41]. Educational attainment serves as a cognitive foundation. The highly educated elderly had stronger information acquisition abilities and more comprehensive vaccine knowledge. They could rationally judge the value of influenza prevention and control and were more willing to get vaccinated. Monthly family income directly constitutes an economic constraint. The elderly with sufficient income have no concerns about vaccine costs and are more willing to take preventive measures.

The VCR is higher among elderly individuals who reported self-care ability compared to those who could not, and it was also higher among those with chronic diseases, consistent with other studies [42]. Elderly people living in larger nursing homes were more likely to be vaccinated, possibly because living with others made them more concerned about contracting the flu, and they could obtain more information about the vaccination from other people, thereby enhancing their awareness and willingness to get vaccinated. This finding is consistent with the results of studies in Shanghai and Beijing [43,44], contrary to the results of a foreign study [45].

The vaccination willingness of the elderly in nursing homes was affected by their self-evaluation of health status, prior experience of influenza-like symptoms, and the environment of the nursing homes. The elderly with poor health status and chronic disease face a higher risk of severe influenza, have a stronger risk perception, and show a more positive willingness to be vaccinated [46,47]. Elderly people who have experienced influenza-like symptoms show a significant increase in their willingness to be vaccinated after experiencing the pain of these symptoms [48]. This study found that the elderly living in suburban nursing homes are more willing to be vaccinated, which is contrary to expected results. It may be that they are more closely connected with suburban neighbors, and word-of-mouth has thus enabled the elderly to obtain more vaccination information. Further, perhaps the elderly in suburban settings are less exposed to online misinformation or negative vaccine narratives, and therefore have a higher trust in vaccines.

The reluctance of the elderly to receive the flu vaccine is mainly due to concerns about adverse reactions, followed by difficulties in accessing the vaccine due to mobility issues or distance, concerns about the quality of the vaccine, and lack of awareness of the vaccine. This highlights the need for greater awareness of flu vaccines among elderly people in nursing homes. The CDC or Community Health Service Centers (CHSCs) should increase awareness through initiatives such as health education campaigns in nursing homes and other key institutions, or by informing residents through telephone or television before the flu season. Furthermore, guided by the concept of integrating medical and preventive services and leveraging existing medical consortia [49], nursing homes should be encouraged to connect with nearby hospitals to establish a green channel for vaccination and medical care for elderly residents in nursing homes.

Nursing homes should enhance the promotion and guidance around the flu vaccine for both staff and residents, and should educate staff, residents, and their families about the importance, safety, and effectiveness of vaccination through special lectures, informative posters, and service reminders. They could also emphasize the effectiveness of vaccination in preventing diseases such as flu and pneumonia.

Secondly, a safe living environment should be provided to reduce the risk of the flu among seniors. Specific measures could be as follows: (1) Environmental management: Regularly clean and disinfect public areas and frequently touched surfaces. Ensure daily ventilation by opening windows. (2) Personal hygiene reinforcement: Provide sufficient hand-washing facilities and encourage staff and residents to wash their hands before eating and after using the restroom. (3) Visitor and health monitoring: During flu seasons, remind the elderly to wear masks when going out and strengthen health monitoring. Meanwhile, prevent individuals with flu-like symptoms such as fever and cough from visiting residents in the nursing home. (4) Nutrition and activity support: Offer a balanced diet with increased protein and vitamin intake to boost immunity in the elderly. Encourage and assist the elderly in performing moderate exercises such as walking or Tai Chi [50]. Concurrently, the above should be effectively communicated in key institutions and village/neighborhood committees through on-site education, newspapers, posters, and service reminders. Finally, nursing homes should actively train staff to educate residents about vaccination and provide a healthy, safe environment.

This study is the first to investigate influenza incidence, vaccination status, and willingness of elderly people to receive vaccination in nursing homes, and is significant for the vaccination of elderly people in collective living environments. In foreign countries, influenza vaccines for the elderly are included in the national immunization program or fully reimbursed by medical insurance. Nursing homes generally provide on-site vaccination services, which makes the VCR of the elderly in nursing homes much higher than that in the Chinese population as a whole. This study provides scientific evidence to inform the development of vaccination strategies and implementation plans for older adults residing in nursing homes in China.

However, the study does have some limitations. The retrospective survey and cross-sectional study design may introduce selection and recall bias during the questionnaire process. Additionally, the research subjects may have overlooked elderly people with unclear speech, unclear thoughts, and caregivers not in the nursing home who are unaware of the situation. This study focused on analyzing objective factors such as demographic characteristics, institutional characteristics, and health status. Nonetheless, it did not explore subjective and external factors, including psychological factors (the underlying psychology of vaccine hesitation), social support (recommendations from institutional medical staff), and policy factors (the potential impact of vaccine subsidies). The study only assessed the situation during the 2024–2025 influenza season. It did not conduct longitudinal tracking of influenza vaccination rates or infection rates among the elderly in nursing homes. As a result, it was impossible to analyze the time trend of vaccination behavior and evaluate the long-term prevention and control effect of vaccination on influenza outbreaks.

Future studies could adopt a multi-center, longitudinal design, expand geographic coverage and sample size, include highly vulnerable groups such as those with severe disabilities and mental illnesses, and conduct in-depth analyses of potential influencing factors such as psychology and policy. This would provide a more comprehensive basis for influenza vaccination strategies in national pension institutions.

## 5. Conclusions

In this study, for the first time, we quantitatively assessed the influenza VCR among elderly individuals in nursing homes in Shanghai, China. The findings revealed that the influenza VCR among the elderly in nursing homes was relatively low, insufficient to establish an immune barrier. Moreover, due to the close proximity in which elderly residents in nursing homes live, they are highly susceptible to clustered influenza outbreaks. The vaccination uptake among the elderly is primarily influenced by attitudes, knowledge, and physical ability to attend a vaccination center. Consequently, it is necessary to intensify publicity efforts aimed at high-risk groups, enhance their understanding of influenza viruses and the influenza vaccine, and develop corresponding vaccination policies.

## Figures and Tables

**Table 1:** Analysis of influencing factors of influenza vaccination and willingness of the elderly in nursing homes (single factor).

	Coverage (*N* = 1024)				Willingness (*N* = 967)			
Variables	Yes	No	χ^2^	*p*-Value	Yes	No	χ^2^	*p*-Value
Total	57 (5.57)	967 (94.43)			149 (16.21)	818 (83.79)		
Sociodemographic								
Gender								
Male	19 (6.19)	288 (93.81)	0.323	0.57	47 (16.32)	241 (83.68)	0.261	0.609 *
Female	38 (5.30)	679 (94.70)			102 (15.02)	577 (84.98)		
Immigration status								
Residents	46 (5.26)	828 (94.74)	1.044	0.307	133 (16.06)	695 (83.94)	1.625	0.202
Migrants	11 (7.33)	139 (92.67)			16 (11.51)	123 (88.49)		
Age (years)								
≤80	14 (6.17)	213 (93.83)	3.485	0.175	38 (17.84)	175 (82.16)	1.892	0.204 *
81–90	36 (6.33)	533 (93.67)			82 (15.38)	451 (84.62)		
>90	7 (3.07)	221 (96.93)			29 (13.12)	192 (86.88)		
Marital status								
Single	18 (6.98)	240 (93.02)	3.006	0.222 *	38 (15.83)	202 (84.17)	0.045	0.978
Married	0	33 (100.00)			5 (15.15)	28 (84.85)		
Divorced or widow	39 (5.32)	694 (94.68)			106 (15.27)	588 (84.73)		
Educational attainment								
Primary school and below	23 (3.48)	637 (96.52)	60.866	<0.001	92 (14.44)	545 (85.56)	1.942	0.585
Middle school	8 (3.77)	204 (96.23)			34 (16.67)	170 (83.33)		
High school	12 (11.88)	89 (88.12)			15 (16.85)	74 (83.15)		
Bachelor’s degree or above	14 (27.45)	37 (72.55)			8 (21.62)	29 (78.38)		
Monthly household income, (CNY) a								
<3000	9 (3.24)	269 (96.76)	38.191	<0.001	35 (13.01)	234 (86.99)	4.004	0.406
3000–4999	21 (5.25)	379 (94.75)			66 (17.41)	313 (82.59)		
5000–8000	17 (17.71)	79 (82.29)			15 (18.99)	64 (81.01)		
>8000	4 (15.38)	22 (84.62)			4 (18.18)	18 (81.82)		
Unknown	6 (2.68)	218 (97.32)			29 (13.30)	189 (86.70)		
Children								
0	3 (4.76)	60 (95.24)	4.212	0.378 *	17 (28.33)	43 (71.67)	9.864	0.043
1	11 (7.05)	145 (92.95)			24 (16.55)	121 (83.45)		
2	22 (7.26)	281 (92.74)			38 (13.52)	243 (86.48)		
3–5	20 (4.21)	455 (95.79)			68 (14.95)	387 (85.05)		
>5	1 (3.70)	26 (96.30)			2 (7.69)	24 (92.31)		
Self-care ability								
No	9 (1.65)	535 (98.35)	33.787	<0.001	82 (15.33)	453 (84.67)	0.006	0.938
Yes	48 (10.00)	432 (90.00)			67 (15.51)	365 (84.49)		
Self-rated health status								
Healthy	20 (8.66)	211 (91.34)	5.958	0.051	38 (18.01)	173 (81.99)	8.172	0.017
Normal	28 (4.38)	612 (95.62)			100 (16.34)	512 (83.66)		
Unhealthy	9 (5.88)	144 (94.12)			11 (7.64)	133 (92.36)		
Underlying disease								
0	10 (6.37)	147 (93.63)	0.423	0.809	19 (12.93)	128 (87.07)	2.256	0.324
1	20 (5.85)	322 (94.15)			45 (13.98)	277 (86.02)		
≥2	27 (5.14)	498 (94.86)			85 (17.07)	413 (82.93)		
Symptoms of influenza								
No	44 (5.76)	720 (94.24)	0.093	0.761	86 (25.51)	634 (74.49)	25.95	<0.001 *
Yes	13 (5.00)	247 (95.00)			63 (11.94)	184 (88.06)		
Nursing home location								
City proper	1 (0.69)	144 (99.31)	8.557	0.014	29 (20.14)	115 (79.86)	10.242	0.006
Rural-urban fringe	21 (7.45)	261 (92.55)			25 (9.58)	236 (90.42)		
Outer suburbs	35 (5.86)	562 (94.14)			95 (16.90)	467 (83.10)		
Nature of nursing homes								
State-owned	15 (4.62)	310 (95.38)	0.819	0.365	58 (18.71)	252 (81.29)	3.815	0.056 *
Private	42 (6.01)	657 (93.99)			91 (13.85)	566 (86.15)		
Nursing home level								
Level 1	10 (3.41)	283 (96.59)	6.224	0.101	46 (16.25)	237 (83.75)	1.552	0.67
Level 2	29 (7.30)	368 (92.70)			60 (16.30)	308 (83.70)		
Level 3	5 (3.65)	132 (96.35)			16 (12.12)	116 (87.88)		
Unrated	13 (6.60)	184 (93.40)			27 (14.67)	157 (85.33)		
Scale of nursing home (number of beds)								
≤150	2 (0.64)	309 (99.36)	38.878	<0.001	47 (15.21)	262 (84.79)	13.96	0.001
151–299	22 (4.94)	423 (95.06)			49 (11.58)	374 (88.42)		
≥300	33 (12.31)	235 (87.69)			53 (22.55)	182 (77.45)		

^a^ 1 US dollar = 7.01 Chinese yuan; * Fisher’s exact test.

**Table 2:** Multivariate logistic regression model of elderly people in nursing homes based on influenza vaccination.

Variables	aOR	95% CI	*p*-Value
Education attainment			
Primary school and below		Ref.	
Middle school	3.800	1.480–9.758	0.006
High school	5.138	1.738–15.191	0.003
Bachelor’s degree or above	1.913	0.711–5.152	0.199
Monthly household income (Chinese yuan)			
<3000		Ref.	
3000–4999	1.241	0.402–3.824	0.707
5000–8000	0.604	0.228–1.599	0.310
>8000	0.216	0.072–0.644	0.006
Unknown	0.283	0.065–1.238	0.094
Self-care ability			
No		Ref.	
Yes	0.269	0.123–0.591	0.001
Nursing home location			
City proper		Ref.	
Rural-urban fringe	2.068	0.179–23.900	0.561
Outer suburbs	0.587	0.305–1.127	0.109
Scale of nursing home (number of beds)			
≤150		Ref.	
151–299	9.033	1.531–53.305	0.015
≥300	2.629	1.359–5.084	0.004

**Table 3:** Multivariate logistic regression model of elderly people in nursing homes based on influenza vaccination willingness.

Variables	aOR	95% CI	*p*-Value
Children			
0		Ref.	
1	0.206	0.042–1.009	0.051
2	0.427	0.092–1.986	0.278
3–5	0.516	0.114–2.341	0.391
>5	0.436	0.098–1.940	0.276
Self-evaluation of health status			
Healthy		Ref.	
Normal	0.331	0.159–0.690	0.003
Unhealthy	0.398	0.204–0.779	0.007
Symptoms of influenza			
No		Ref.	
Yes	2.730	1.861–4.007	<0.001
Nursing home location			
City proper		Ref.	
Rural-urban fringe	0.711	0.398–1.273	0.251
Outer suburbs	1.537	1.099–2.941	0.020
Scale of nursing home (number of beds)			
≤150		Ref.	
151–299	1.991	1.154–3.435	0.013
≥300	2.158	1.374–3.390	0.001

## Data Availability

Restrictions apply to the availability of these data. Data were obtained from the Shanghai Pudong New Area Center for Disease Control and Prevention (Shanghai Pudong New Area Health Supervision Institute) (PDCDC) and are available from the authors with the permission of PDCDC.

## Abbreviations

The following abbreviations are used in this manuscript:

China CDC	Chinese Center for Disease Control and Prevention
NIP	China’s National Immunization Program
VCR	Vaccination Coverage Rate
PDCDC	Shanghai Pudong New Area Center for Disease Control and Prevention
SIPIS	Shanghai Immunization Planning Information System
ORs	Odds Ratios
CIs	Confidence Intervals
WHO	The World Health Organization
CHSCs	Community Health Service Centers
